# The Methods of Bacteriological Investigation

**Published:** 1886-10

**Authors:** 


The Methods of Bacteriological Investigation. *By* Dr. Ferdinand Hueppe, *Docent in Hygiene and Bacteriology in the Chemical Laboratory of R. Fresenius at Wiesbaden. Translated by* Hermann M. Biggs, m. d. *Octavo, pp. 218. New York:* D. Appleton & Co. *Chicago:* A. C. McClurg & Co.

The translator was induced to present the work of Dr. Hueppe to the American public because of the want in English of a satisfactory text-book for the students working under his direction in the Carnegie Laboratory.

Of theoretical and polemical treatises on bacteriology there is no lack, many of them having been written by those who do not understand the first principles of bacteriology, and who could not give any idea of the relative value of solid and fluid culture-media. To such the careful study of this volume might prove advantageous.

The work is written largely from the standpoint of the Berlin School by a pupil of Geheimrath Koch, and presents many of his peculiar views and methods. It is divided into seven parts respectively: 1. Spontaneous generation and the principles of sterilization. 2. Forms of bacteria and microscopical technique. 3. Culture-methods. 4. Inoculations for the determination of the casual relation of bacteria-growth to decomposition and disease. 5. General biological problems. 6. Special hygienic investigation. 7. Bacteriology as an object of instruction.

As a text-book it is clear and concise, and we trust will do much to stimulate the study of this important branch of biological research in this country. The English of the book could in many cases be criticised, but one familiar with the curt idioms of the original will readily overlook this.

